# Beyond the Bench: Keeping Kids’ Environments Safe

**Published:** 2006-04

**Authors:** Tanya Tillett

A safe home environment is the first line of defense in protecting children’s health. And since many kids spend a great amount of time in schools and child care facilities, safeguarding their health in those places is also essential. The Community Outreach and Education Core (COEC) of the Environmental Health Sciences Center in Molecular and Cellular Toxicology with Human Applications at Wayne State University, in collaboration with the Detroit Head Start and the EPA, has developed the Healthy Homes = Healthy Kids Train-the-Trainer Program to ensure that parents and other caregivers receive information that allows them to create a hazard-free atmosphere for kids. These participants can then pass information along to other parents and caregivers. By training the trainer, the program ensures that a larger number of teachers and caregivers, and ultimately, children can be effectively reached and introduced to the basic concepts of environmental health science.

Participants in the workshops are introduced to common household safety issues including toxic heavy metals (such as lead and mercury), indoor air pollutants (such as mold, asthma triggers, and combustion by-products), drinking water quality, food safety, pest control, and poisonous substances (such as medications, perfumes, and dish detergents) that children could mistake for food or drink.

Next, the participants learn about the health effects of these hazards, and are taught healthy practices to follow in the home, available methods for detecting and screening for poisonings, and ways to tap into additional local, state, and national resources. The program also provides hands-on activities for the participants that let them practice reacting to possible hazardous situations, such as finding a child playing with a toxic household cleaner.

The content of each workshop covers about six hours, but can be broken up over several days if needed. Program developers use pre- and post-tests to measure knowledge gained by participants, and also conduct follow-up surveys to gauge the effectiveness of the program and track how much the information is being used by workshop attendees. In addition to hands-on training, the program also provides a poster that highlights the hazards discussed in the sessions and a variety of fact sheets detailing common environmental hazards and prevention strategies. The fact sheets and poster are all available in English, Spanish, and Arabic.

The COEC recognizes the importance of community partnerships in providing effective outreach initiatives, and credits its outreach partners with helping keep the program responsive and successful. Train-the-trainer workshops have been conducted at Head Start in Detroit and the nonprofit Child Care Coordinating Council of Detroit/Wayne County.

“The COEC, through interactions and input from community groups . . . utilizes the scientific knowledge and research of center members to provide assistance to the community through education, prevention, and resource identification,” says COEC project coordinator Lisa Pietrantoni.

Materials for the program are continuously updated as new information becomes available, and new topics are currently being developed. A total of 174 caregivers have been trained so far, and additional training sessions are scheduled for this spring.

## Figures and Tables

**Figure:**
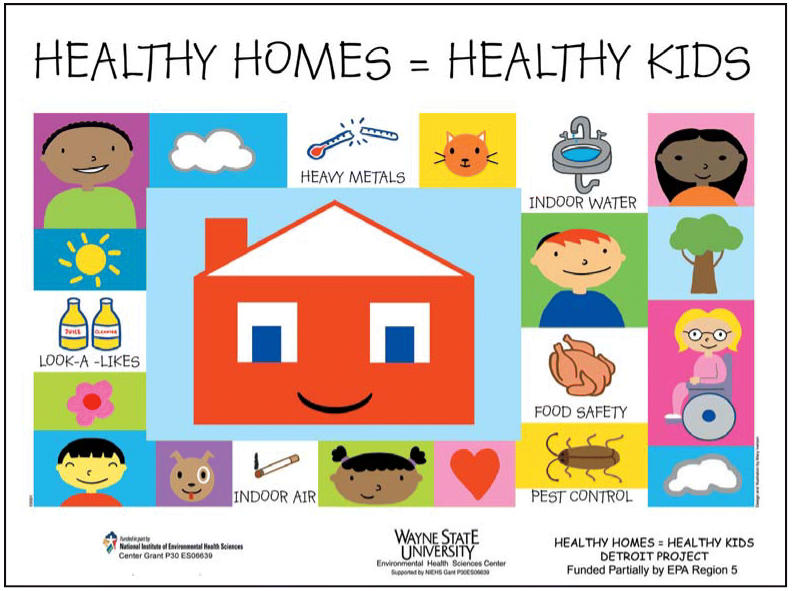
Taking better care of kids. The Healthy Homes = Healthy Kids Train-the-Trainer Program was developed to introduce parents and caregivers to environmental hazards in children’s indoor environments and provide them with information on how to avoid such hazards and improve children’s health.

